# Cryotrapping peroxide in the active site of human mitochondrial manganese superoxide dismutase crystals for neutron diffraction

**DOI:** 10.1107/S2053230X21012413

**Published:** 2022-01-01

**Authors:** Jahaun Azadmanesh, William E. Lutz, Leighton Coates, Kevin L. Weiss, Gloria E. O. Borgstahl

**Affiliations:** a Eppley Institute for Research in Cancer and Allied Diseases, University of Nebraska Medical Center, 986805 Nebraska Medical Center, Omaha, NE 68198-6805, USA; b Second Target Station, Oak Ridge National Laboratory, 1 Bethel Valley Road, Oak Ridge, TN 37831, USA; c Neutron Scattering Division, Oak Ridge National Laboratory, 1 Bethel Valley Road, Oak Ridge, TN 37831, USA

**Keywords:** human manganese superoxide dismutase, neutron diffraction, large unit cell, cryotrapping, peroxide

## Abstract

Structurally identifying the enzymatic intermediates of redox proteins has been elusive due to difficulty in resolving the H atoms involved in catalysis and the susceptibility of ligand complexes to photoreduction from X-rays. Cryotrapping ligands for neutron protein crystallography combines two powerful tools that offer the advantage of directly identifying hydrogen positions in redox-enzyme intermediates without radiolytic perturbation of metal-containing active sites. However, translating cryogenic techniques from X-ray to neutron crystallo­graphy is not straightforward due to the large crystal volumes and long data-collection times. Here, methods have been developed to visualize the evasive peroxo complex of manganese superoxide dismutase (MnSOD) so that all atoms, including H atoms, could be visualized. The subsequent cryocooling and ligand-trapping methods resulted in neutron data collection to 2.30 Å resolution. The *P*6_1_22 crystal form of MnSOD is challenging because it has some of the largest unit-cell dimensions (*a* = *b* = 77.8, *c* = 236.8 Å) ever studied using high-resolution cryo-neutron crystallography. The resulting neutron diffraction data permitted the visualization of a dioxygen species bound to the MnSOD active-site metal that was indicative of successful cryotrapping.

## Abstract 2

Human mitochondrial manganese superoxide dismutase (MnSOD) is a major player in alleviating oxidative stress within the mitochondria, which is a characteristic of a wide range of human diseases. Here, the methods leading to the structural visualization of the evasive peroxo complex of MnSOD by integrating cryotrapping and neutron protein crystallography are described.

## 1. Introduction

Neutron protein crystallography (NPC) is an emerging tool for identifying hydrogen positions in biological macromolecules at modest resolutions (<2.5 Å) due to the neutron scattering of deuterium and hydrogen being on a par with those of carbon, nitrogen and oxygen. Unlike X-rays, neutrons do not affect the electronic states of metals and metal-bound ligands (Carugo & Djinovic Carugo, 2005 ▸). These characteristics of NPC permit a structural association to be made between the oxidation state of the metal centre of an oxidoreductase and the protonation status of the active site. We have previously developed methods that have led to neutron data collection from manganese superoxide dis­mutase (MnSOD) crystals to a resolution at which hydrogen positions could be seen (Azadmanesh, Trickel, Weiss *et al.*, 2017 ▸). This was particularly challenging for our crystal system owing to the large unit-cell edge approaching 240 Å. A multipronged approach was used to circumvent these issues. Firstly, perdeuterated (fully deuterium-labelled) MnSOD protein was required. Deuterium has a 40-fold lower incoherent neutron scattering compared with hydrogen and leads to a significant improvement in diffraction signal (O’Dell *et al.*, 2016 ▸). Secondly, diffraction is weakened by the low flux of the neutron beam, so the MnSOD crystal needed to be grown to a large volume. Thirdly, a time-of-flight neutron diffractometer was required due to the large unit-cell axes (Blakeley, 2011 ▸). The MaNDi diffractometer at Oak Ridge National Laboratory (ORNL) utilizes neutrons between 2 and 4 Å wavelength that can be resolved using the neutron time of flight. The Laue diffraction pattern can then be broken into monochromatic slices, which reduces the number of reflections that are harmonically or spatially overlapped and also increases the signal to noise (Langan *et al.*, 2008 ▸; Coates *et al.*, 2010 ▸). With these methods in hand, room-temperature neutron diffraction data can be routinely collected from MnSOD crystals to sufficient resolutions (2.2–2.3 Å) that hydrogen positions can be observed.

Human mitochondrial MnSOD is a heavily studied oxidoreductase in clinical research due to its central role in the oxidative stress response (Miriyala *et al.*, 2012 ▸). Like other oxidoreductases, MnSOD uses concerted proton–electron transfer (CPET) reactions for catalysis. The CPETs convert 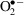 into either oxygen (O_2_; *k*_1_) or hydrogen peroxide (H_2_O_2_; *k*_2_), depending on the oxidation state of the metal and the protonation status of the active site (Holm *et al.*, 1996 ▸). The CPET mechanisms of most oxidoreductases are not well defined due to the difficulty in discerning the proton transfers that coincide with each electron transfer. For MnSOD, we previously utilized neutron protein crystallography (NPC) to identify the changes in protonation at the active site between the trivalent and divalent states of the active-site manganese ion (Azadmanesh, Lutz, Weiss *et al.*, 2021 ▸). This work indicated that the mechanism of MnSOD utilizes an array of proton transfers for each electron transfer, which include cyclic deprotonation and protonation of the active-site glutamine. 

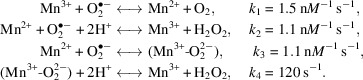




Among all SODs, MnSOD exhibits the unique feature of product inhibition 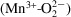 that limits its output of hydrogen peroxide (*k*_3_ and *k*_4_), adding another layer of complexity to its CPET mechanism (Hearn *et al.*, 2001 ▸). Human MnSOD has the greatest product inhibition among studied homologs and contrasts with MnSODs from other eukaryotic organisms such as *Saccharomyces cerevisiae*, which have negligible amounts (Barnese *et al.*, 2010 ▸). MnSOD instigates product inhibition from the Mn^2+^ redox state 50% of the time during steady-state conditions due to the equal *k*_2_ and *k*_3_ rates. The inhibited complex is thought to be a peroxide anion 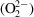 or protonated derivative, bound at the active site 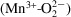 , that is slowly released by an alternative configuration of proton transfers (*k*_4_) that differ from the un­inhibited reaction cycle of *k*_1_ and *k*_2_ (Hearn *et al.*, 1999 ▸). However, the true natures of the product-inhibited active site and the proton transfers relieving inhibition have not yet been discerned.

In this work, we took neutron diffraction data collection of MnSOD a step further by developing ligand-cryotrapping methods for the product-inhibited state. Cryogenic cooling of large crystals for neutron diffraction was prevalent as early as 2004 (Blakeley *et al.*, 2004 ▸) and such methods require a further extent of precision due to the susceptibility of larger crystals to ice buildup (Kwon *et al.*, 2018 ▸). For MnSOD, a further challenge was present due to the large unit-cell axes of the crystal form, the goal of trapping an enzymatic intermediate and the need for the nearly complete replacement of hydrogen with deuterium. For deuterium exchange, we used a simple process in which the labile hydrogen positions in a large MnSOD crystal could be exchanged to ∼85% deuterium within 2 h by gentle equilibration with a deuterated cryoprotectant. Several parameters were optimized to ensure successful cryocooling with a D_2_O_2_ soak while maintaining diffraction quality over 14 days of neutron data collection. The Trp161Phe MnSOD variant was utilized as it enriches the product-inhibited state and maintains hydrogen-bond interactions at the active site (Cabelli *et al.*, 1999 ▸). This led to neutron data collection to 2.30 Å resolution, with preliminary maps demonstrating the binding of a putative D_2_O_2_ derivative species to the active site at full occupancy. For oxidoreductases, the work presented is noteworthy because it demonstrates the steps leading to the visualization of a paramagnetic centre–oxygen species interaction without radiation-induced perturbations.

## 2. Materials and methods

### 2.1. Expression

Detailed methods for the recombinant expression of deuterated MnSOD at the ORNL Bio-Deuteration Laboratory, purification and crystallization have been described previously (Azadmanesh, Trickel, Weiss *et al.*, 2017 ▸). In brief, large-scale expression of perdeuterated Trp161Phe MnSOD in *Escherichia coli* used D_2_O minimal medium with d_8_-glycerol as the carbon source (Meilleur *et al.*, 2009 ▸). NaOD was added on demand to maintain a pD value of >7.3 (where pD is the measured pH + 0.4) and cell-strain fidelity was maintained with 100 µg ml^−1^ kanamycin. Induction was performed at 37°C with isopropyl β-d-1-thiogalactopyranoside (IPTG) at a final concentration of 1 m*M* and coincided with the addition of 1.4 g l^−1^ MnCl_2_ for metal incorporation (Whittaker & Whittaker, 2012 ▸). Induction occurred at an OD_600_ of 7.4, and cells were harvested at an OD_600_ of 18.0 by centrifugation, yielding 41 g of deuterated cell paste.

### 2.2. Crystallization

Perdeuterated Trp161Phe MnSOD crystals were grown in Granada Crystallization Boxes (GCBs, Triana) through capillary counterdiffusion using fused quartz capillary tubes (VitroCom) that had inner diameters of 2.0 mm and outer diameters of 2.4 mm (Ng *et al.*, 2003 ▸). The crystal utilized for data collection was grown from a capillary filled with 25 mg ml^−1^ protein that was plugged with 40 mm 2%(*w*/*w*) agarose and inserted into a GCB subsequently filled with precipitating agent consisting of 4 *M* potassium phosphate pH 7.8. The pH of the phosphate buffer was achieved using a 91:9 ratio of K_2_HPO_4_:KH_2_PO_4_. The GCBs were delivered to the International Space Station by SpX-17 as part of the Perfect Crystals NASA payload and were returned after one month on SpX-18. The crystals within GCBs were observed to be resilient against travel damage and were placed within carry-on baggage during further aircraft travels to the Omaha Structural Biology Core Facility and to ORNL. It should be noted that Earth-grown large-volume crystals have sufficient diffraction quality (Azadmanesh, Trickel, Weiss *et al.*, 2017 ▸) and microgravity-grown crystals were used here due to availability and convenience.

### 2.3. Deuterium exchange

**Figure 1:**
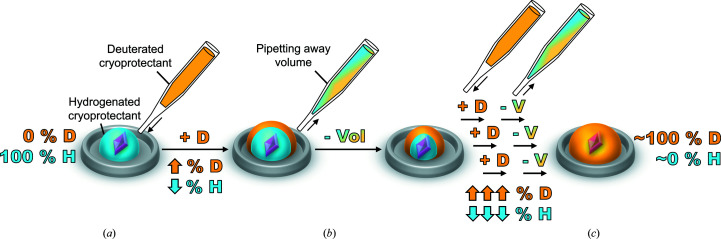
A simple and gentle method for H/D exchange for cryogenic neutron crystallography. (*a*) Start with a crystal immersed in a hydrogenated cryoprotectant. A deuterated counterpart is then added to the solution with gentle mixing, which results in the percentage of deuterated content increasing while the percentage of hydrogenated content decreases. Initially, modest volumes of deuterated cryoprotectant should be added to minimize damage to the crystals. (*b*) In preparation for further dilution of hydrogenated content, the volume of the solution is decreased. (*c*) Iterations of processes (*a*) and (*b*) lead to the near-complete depletion of hydrogenated solution and immersion of the crystal in nearly 100% deuterated cryoprotectant.

From X-ray analysis, we found that MnSOD crystals could be cryocooled by increasing the concentration of the phosphate precipitating agent (Azadmanesh, Trickel, Weiss *et al.*, 2017 ▸). Thus, we planned to immerse crystals in 4 *M* deuterated potassium phosphate (K_2_DPO_4_:KD_2_PO_4_) pD 7.8 (calculated by adding 0.4 to the measured pH reading) to serve as a cryoprotectant that also promoted H/D exchange and minimized the incoherent scattering of H atoms. However, the crystals were susceptible to cracking when immediately exposed to deuterated solutions and required a precise and gradual exposure. Firstly, prospective crystals for data collection were extracted from the capillaries of the GCBs and placed into a nine-well glass plate with 1 ml hydrogenated 4 *M* potassium phosphate pH 7.8. Deuterated cryoprotectant was first introduced into the solution containing the crystal by the addition of a 0.1 ml volume with gentle mixing. After 2 min, the process was repeated until a total volume of 0.5 ml had been added to the drop. The net result was a gradual and gentle exposure of the crystal to a deuterated solution to reduce the hydrogen content of the final solution (Fig. 1 ▸ *a*). In this way, the hydrogen was capable of being exchanged away by then removing 0.5 ml of the total solution composed of the mixed hydrogen and deuterium reagents (Fig. 1 ▸ *b*) and performing another series of five additions of 0.1 ml deuterated cryoprotectant. By iterating the process of adding deuterium reagents and pipetting away mixed hydrogenated/deuterated solution over the course of 2 h, the crystal was eventually soaking in a solution composed of entirely deuterated cryoprotectant (Fig. 1 ▸ *c*).

### 2.4. Addition of D_2_O_2_ and cryocooling

**Figure 2:**
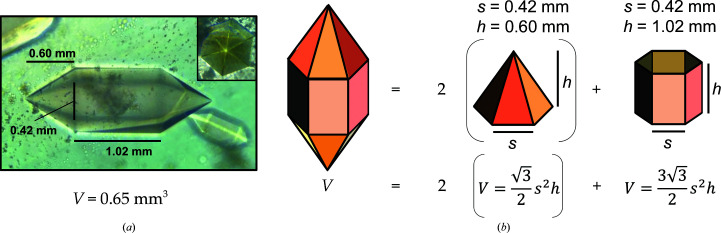
Volume determination and data acquisition of a perdeuterated Trp161Phe MnSOD crystal. (*a*) Image and dimensions of the crystal. The inset shows the six-face morphology when looking down its length. (*b*) Volume calculations of the crystal using the dimensions from (*a*).

**Figure 3:**
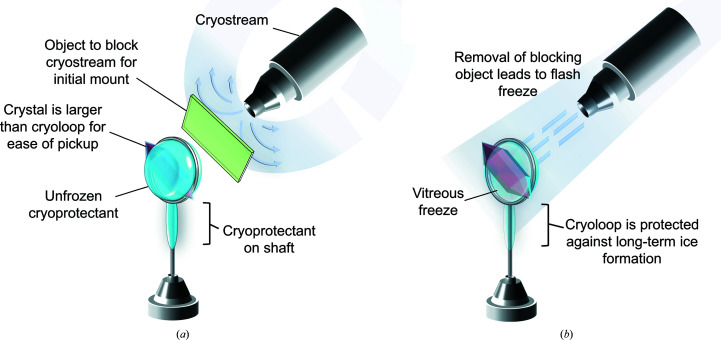
Schematic of the flash-freezng process for large crystals and its key points. (*a*) Material setup prior to flash-freezing. Care should be taken in choosing an appropriate cryoloop diameter and in ensuring that both the crystal and a large portion of the cryoloop stem are well coated in cryoprotectant. In preparation for freezing, the crystal-containing cryoloop is mounted on the goniometer while an object (card) blocks the cryostream. (*b*) After mounting, quick removal of the card leads to a vitreous flash-freeze with the absence of crystalline ice.

After the prospective crystal samples had been immersed in ∼100% deuterated cryoprotectant, D_2_O_2_ was added to the cryoprotectant solution to a final concentration of 1%(*v*/*v*). During 5 min of D_2_O_2_ soaking, a 1 mm diameter cryoloop was well submerged in the deuterated cryoprotectant to prevent the formation of crystalline ice during data collection. After the 5 min soak, a 0.65 mm^3^ crystal (Figs. 2 ▸ *a* and 2 ▸ *b*) was harvested from the deuterated cryoprotectant with the cryoloop and flash-cooled with an Oxford diffraction cryostream system at the MaNDi beamline located at ORNL (Kwon *et al.*, 2018 ▸). Vitreous freezing was performed by blocking the cryostream with a card-like object, placing the crystal-containing cryoloop onto the goniometer and then quickly removing the object blocking the stream (Fig. 3 ▸).

### 2.5. Neutron data collection

Time-of-flight neutron diffraction data were collected to 2.30 Å resolution at 100 K from a 0.65 mm^3^ perdeuterated crystal using the MaNDi instrument at ORNL (Table 1 ▸; Coates *et al.*, 2010 ▸, 2015 ▸). All neutrons between 2 and 4 Å wavelength were used and were discerned by time of flight. The crystal was held static for each image but was rotated 10° in φ between images for a total of 12 images. Data were integrated using the 3D profile-fitting algorithm (Sullivan *et al.*, 2018 ▸) from the *MANTID* software package (Arnold *et al.*, 2014 ▸) and wavelength-normalized and scaled with *LAUENORM* from the Daresbury Laue Software Suite (Campbell, 1995 ▸; Helliwell *et al.*, 1989 ▸; Arzt *et al.*, 1999 ▸; Campbell *et al.*, 1998 ▸; Hao *et al.*, 2021 ▸). Data reduction yielded a data set with 99% completeness.

### 2.6. X-ray data collection

X-ray diffraction data were collected to 1.87 Å resolution using a Rigaku FR-E SuperBright home source from a separate perdeuterated crystal without peroxide soaking (Table 1 ▸). Data collection was at 100 K with 4 *M* potassium phosphate pH 7.8 as the cryoprotectant. The crystal was rotated 0.5° along the φ axis during each frame of data collection. Data were reduced using *HKL*-3000 for indexing, integration and scaling (Minor *et al.*, 2006 ▸).

### 2.7. Preliminary refinement

Preliminary refinement of the neutron model was performed with *phenix.refine* from the *Phenix* suite (Liebschner *et al.*, 2019 ▸). The models were refined separately due to the known alterations in solvent and metalloprotein structure induced by X-rays that are not present with neutrons (Garman, 2010 ▸), and joint refinement consistently led to higher *R*_work_ and *R*_free_ statistics. The higher resolution X-ray model was initially refined against the corresponding data set using PDB entry 5vf9 for simple molecular replacement (Azadmanesh, Trickel & Borgstahl, 2017 ▸). The resulting X-ray coordinates were used as the starting model for preliminary neutron refinement, where backbone torsion restraints were derived from the X-ray model and implemented in refinement of the neutron model with *phenix.refine* (Afonine *et al.*, 2012 ▸). The preliminary refinement data are available at https://doi.org/10.5281/zenodo.5715407.

### 2.8. Determination of backbone amide H/D content

The extent of backbone amide deuterium exchange was estimated for the neutron data set using the following refinement process for the neutron model.

(i) Initially, all nonprotein entities were excluded from the starting model, while only D atoms at non-exchangeable sites were included. For several iterations, protein atoms were manually fitted into |*F*_o_| − |*F*_c_| difference neutron scattering-length density with *Coot* and refined (Emsley *et al.*, 2010 ▸).
(ii) Next, for each residue, the amide N atom of the protein backbone was inspected for nearby |*F*_o_| − |*F*_c_| neutron scattering-length peaks reflecting the position of the amide proton. Since the neutron scattering length of deuterium is positive (6.671 fm) while that of hydrogen is negative (−3.741 fm), it is expected that positive |*F*_o_| − |*F*_c_| density indicates the presence of deuterium (O’Dell *et al.*, 2016 ▸; Schröder & Meilleur, 2020 ▸). A lack of density thus indicates a mixture of hydrogen and deuterium due to density cancellation. Following these principles, positive |*F*_o_| − |*F*_c_| peaks at 2.5σ seen at the amide proton position were modelled as deuterium at full occupancy.
(iii) After iterations of positive |*F*_o_| − |*F*_c_| density inspection with deuterium modelling and refinement, the backbone deuterons modelled at full occupancy were validated by performing tests of H/D-occupancy refinement. If several rounds of test refinements led to deuterium occupancies of less than 0.95, the site was left as mixed H/D, whereas sites at 0.95 or higher were reverted to full D occupancy.
(iv) This entire process of inspecting for positive |*F*_o_| − |*F*_c_| difference neutron scattering-length density at the protein backbone, modelling deuterium and validating sites of full D occupancy was repeated until there were no positive |*F*_o_| − |*F*_c_| peaks at the backbone amides. At this point, the remainder of the amide backbone without deuterons/protons was modelled with mixed H/D and the occupancy was refined.

## 3. Results and discussion

### 3.1. Deuterium exchange

Replacing labile H atoms with deuterium is beneficial to both the diffraction quality and the nuclear scattering-length density maps used for model building. With deuterium exchange, the incoherent scattering section at the atom position is decreased 40-fold and leads to large improvements in signal to noise during diffraction when many H atoms are replaced (Blakeley, 2008 ▸). Removing hydrogen also minimizes density cancellation when interpreting maps for model building. Otherwise, the opposite signs of coherent neutron scattering length for the atoms (D, 6.671 fm; H, −3.741 fm) can lead to a lack of density that hinders efforts in all-atom structure solution. Therefore, an effort is made to ensure that crystalline samples are absent of hydrogen, especially at catalytically relevant positions.

For NPC, crystals grown in a hydrogenated environment have been reported to undergo deuterium exchange over weeks to years (Dajnowicz *et al.*, 2017 ▸; Azadmanesh *et al.*, 2018 ▸). A noteworthy example is aspartate aminotransferase, which underwent exchange for approximately one year and had 85% deuteration at all labile positions and 81% at backbone amide proton positions (Dajnowicz *et al.*, 2017 ▸). The extent of deuterium exchange in crystals with regard to time frame is unclear, although it is expected to depend on the manner of exchange, such as vapour diffusion in a sealed environment versus a direct soak, and the solvent:protein ratio of the sample. However, directly soaking the crystals is not preferred as ‘deuterium shock’ leads to a degradation in diffraction quality, presumably due to a lack of acclimatizaton to the new environment (Kwon *et al.*, 2018 ▸). In the present work, we tested a hydrogen-diluting technique (Fig. 1 ▸) that gradually increased the deuterium content of a solution encompassing a crystal over several hours.

**Figure 4:**
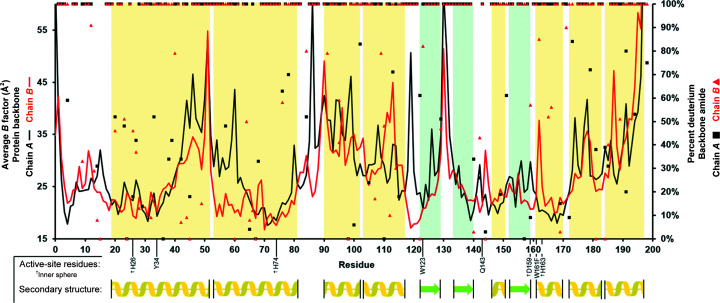
Per-residue plot detailing the average *B* factor of the protein backbone (solid lines) and the fraction of deuterium found at the amide proton position (scatter plot). All data were obtained from preliminary crystallographic refinement as noted in Section 2. Yellow and green areas correspond to the secondary structure noted schematically below the *x* axis. Inner- and outer-sphere active-site residues are indicated along the *x* axis, where inner-sphere residues are indicated by a dagger.

Since the crystal sample was grown in hydrogenated solution, the extent of H/D exchange at labile positions using a hydrogen-diluting method (Fig. 1 ▸) over the 2 h period could be measured with crystallographic data. The contrasting neutron scattering lengths between deuterium (6.671 fm) and hydrogen (−3.741 fm) permitted the fraction of H/D occupying the position of each amide proton to be estimated with careful steps of model building and H/D-occupancy refinement (Fig. 4 ▸). In total, the asymmetric unit composed of a dimer had a deuterium content of 83% for the amide backbone. Areas with less than 50% deuterium exchange for the amide backbone coincide with the presence of secondary structure and/or backbone *B*-factor values less than the average of 29 Å^2^ (Fig. 4 ▸). The extent of exchange in these areas is expected and reflects exchange between the amide proton and D_2_O being limited by (i) the amide group participating in hydrogen bonding with other parts of the protein and therefore being less amenable to amide-solvent proton transfer and/or (ii) limited solvent access, where low *B*-factor values may reflect well ordered portions of the backbone. Calculating the fraction of deuterium exchange for areas of the protein that do not participate in secondary structure leads to a deuterium content of 88%. Similarly, calculating the fraction of deuterium only for residues with *B*-factor values of less than the average of 29 Å^2^ yields a backbone deuterium content of 89%. In general, the amide backbone was well exchanged during the 2 h deuterium soak of the crystal, including at ordered regions, and it can be expected that close to full deuterium exchange occurs in catalytically relevant areas that are typically exposed to solvent.

While exchange was appreciable for the present MnSOD *P*6_1_22 crystal system, four factors should be considered on application to other crystal systems.

(i) Firstly, it can be expected that the solvent content of the crystal and the size of the unit cell will affect the speed of deuterium exchange. For the human Trp161Phe MnSOD crystal from which neutron diffraction data were collected (Table 1 ▸), the solvent content is 47.2% as predicted by the Matthews coefficient (Weichenberger *et al.*, 2015 ▸), with a unit-cell volume of 1 241 285 Å^3^.
(ii) Secondly, exchange among asymmetric subunits may not be equal due to the nature of crystal lattice contacts. For the *P*6_1_22 form of MnSOD, chain *B* is more solvent-exposed than chain *A* (Azadmanesh, Lutz, Coates *et al.*, 2021 ▸) and consequentially had ∼5% greater deuterium exchange when measured by our preliminary refinements.
(iii) Thirdly, it may not be feasible to reach 100% deuteration at labile positions, as illustrated by the diffraction data for aspartate aminotransferase that underwent exchange for about one year (Dajnowicz *et al.*, 2017 ▸). Even in the cases of ascorbate peroxidase and T4 lysozyme crystallized with deuterated reagents, the deuterated content at the amide backbone for either did not exceed 82% (Kwon *et al.*, 2016 ▸; Li *et al.*, 2017 ▸). However, differences in refinement strategy may partially account for these differences.
(iv) Fourthly, whether the solvent is accessible to the catalytically relevant areas of the protein (or other areas of interest) should be considered.

There is no well established protocol on how to perform H/D-occupancy refinement, and differences in strategy are seen in the literature. This is complicated by the negative neutron scattering-length density of hydrogen that is not easily distinguished in density maps above 2.0 Å resolution. Density cancellation and the incoherent scattering cross-section impede visualization of its density. The issue was tackled in the work on aspartate aminotransferase by initially modelling all labile hydrogen positions as deuterium. Deuterium is then occupancy refined in the range of −0.56 to 1.00 since the scattering length of hydrogen is −0.56 times that of deuterium (Dajnowicz *et al.*, 2017 ▸). Afterwards, the position is converted to the presence of both H and D atoms with positive occupancies that sum to 1.0. In comparison, several refinement packages introduce H and D at 0.50 occupancy in the starting model (Schröder & Meilleur, 2020 ▸). Other factors that may affect H/D-occupancy determination are the magnitude of the *B*-factor restraints, the completeness of the neutron data set, model bias, the quality of phasing and the temperature during data collection.

### 3.2. Cryocooling

Cryocooling crystals for NPC is considered to be more challenging than that for X-ray protein crystallography (Coates *et al.*, 2014 ▸). The larger volume of the crystals and the longer data-collection times needed for NPC increase the potential for ice buildup. In addition to making an effort to ensure that the sample is well acclimatized to the cryoprotectant, the cryoloop intended to hold the crystal should be well coated with cryoprotectant along the shaft (Fig. 3 ▸). The size of the loop also contributes to the success of cryocooling. In the case of experiments utilizing a cryoprotectant where deuterated versions are not available, minimizing the size of the loop will lessen the volume of hydrogenated vitreous liquor contributing to the experimental background (Blakeley, 2008 ▸). However, too small a loop may dislodge the crystal. In general, it is advised to use a loop size a little larger than the volume of the crystal (Kwon *et al.*, 2018 ▸). In the present work, the crystal was elongated in shape and measured ∼2.2 mm in length and ∼0.6 mm in width and depth. A 1 mm diameter was utilized where the length of the crystal hung over the circumference of the loop (Fig. 3 ▸). Due to the size of the sample and the high viscosity of the deuterated cryoprotectant, 4 *M* potassium phosphate pD 7.8, the crystal was able to be centred on the loop and picked up within a large drop of liquor without a microscope.

There appears to be a preference in the manner of vitreous freezing in past work. As an example, investigations of T4 lysozyme and Toho1 β-lactamase used a liquid-nitrogen bath (Li *et al.*, 2017 ▸; Kwon *et al.*, 2018 ▸), while the work on ascorbate peroxidase used directly cryocooling on the instrument cryostream similar to our work (Fig. 3 ▸; Kwon *et al.*, 2016 ▸). In the case of the Toho1 β-lactamase crystals, the authors mention that direct cryocooling was feasible, but crystals were lost in the process. Instead, they note that immersion into a dewar overfilled with liquid nitrogen was the most reproducible technique. For cryocooling MnSOD crystals, we found cryocooling on the instrument cryostream to be the most successful technique due to the minimized need for handling. Crystals were able to be picked up from solution, placed on the gonio­meter and cryocooled in seconds without a microscope.

### 3.3. Metal-bound peroxide

Structural analysis of a cryocooled dioxygen species in the active site of an oxidoreductase is particularly challenging with X-rays. For example, of the 11 structures published in the PDB since January 2020 containing a PEO ligand indicating peroxide (PDB entries 7cit, 7kqu, 6lqw, 6k0f, 6k0e, 7ciy, 6lf7, 7dn7, 6lrn, 7dn6 and 7dlq), seven had the ligand either refined with partial occupancy or with appreciably higher *B* factors compared with the rest of the protein. Of the structures with a paramagnetic metal centre adjacent to the PEO molecule, such as iron, copper or zinc, the occupancy of PEO did not exceed 0.64. For MnSOD, there is a cryocooled structure from *E. coli*, PDB entry 3k9s, with PEO bound to the metal with 0.50 occupancy (Porta *et al.*, 2010 ▸). A combination of photoreduction of the metals and ionization of oxygen species may explain these observations. X-rays are known to photoreduce paramagnetic metals and may perturb oxygen species binding to the metal if the binding is dependent on the oxidation state of the metal (Carugo & Djinovic Carugo, 2005 ▸). While oxygen species and their radicals generated by radiolysis, such as 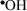 , are considered to be immobilized at temperatures below 110 K, X-ray-induced structural changes have been observed at cryotemperatures for areas that are sensitive to photoreduction (Garman, 2010 ▸). Retinal-bound and metal-containing proteins have several well documented examples (Takeda *et al.*, 2004 ▸; Ravelli & Garman, 2006 ▸). Therefore, the nonreactivity of neutrons to paramagnetic centres provides the opportunity to investigate metal–oxygen complexes such as that of the Mn-peroxo complex of MnSOD.

For NPC, we used the Trp161Phe variant of human MnSOD because of its long-lived inhibited complex as shown by kinetic studies (Hearn *et al.*, 2001 ▸). The inhibited complex is presumed to be a dioxygen species with an unknown protonation state bound to the manganese, but it has not been possible to experimentally investigate it without the perturbations of X-rays. Interestingly, excesses of H_2_O_2_ have been found to induce formation of the complex (Hearn *et al.*, 1999 ▸). Therefore, D_2_O_2_ soaking of Trp161Phe MnSOD was pursued by neutron data collection.

**Figure 5:**
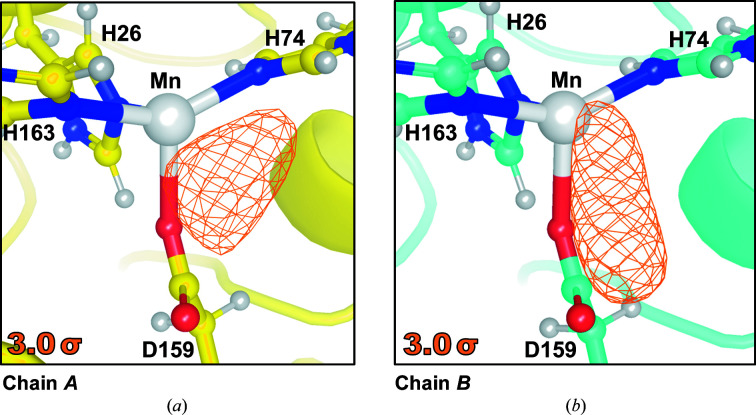
Omit |*F*_o_| − |*F*_c_| difference neutron scattering-length density at 3.0σ indicating different molecular species coordinating to the active-site manganese between chains. (*a*) Omit |*F*_o_| − |*F*_c_| density indicates a triangle-shaped DOD molecule bound to the manganese in chain *A* of the dimeric asymmetric unit. (*b*) Omit |*F*_o_| − |*F*_c_| density indicates a linear-shaped peroxide-derived molecule bound to the manganese in chain *B*. The preliminary refinement data are available at https://doi.org/10.5281/zenodo.5715407.

Investigation of the preliminary |*F*_o_| − |*F*_c_| difference neutron scattering-length density at 3.0σ for the D_2_O_2_-soaked Trp161Phe MnSOD neutron data set reveals that the two subunits of the asymmetric unit have two different species bound to the manganese (Fig. 5 ▸). This is often observed for ligand-bound MnSOD in the *P*6_1_22 crystal form because the active site of chain *B* is more solvent-accessible compared with chain *A* (Azadmanesh, Lutz, Weiss *et al.*, 2021 ▸; Azadmanesh, Trickel & Borgstahl, 2017 ▸). The triangle-shaped density in chain *A* (Fig. 5 ▸ *a*) has been previously seen in room-temperature neutron structures of wild-type Mn^2+^SOD and reflects the presence of a DOD molecule (Azadmanesh, Lutz, Coates *et al.*, 2021 ▸). In contrast, the |*F*_o_| − |*F*_c_| density seen in the more solvent-accessible chain *B* is elongated and appreciably different (Fig. 5 ▸ *b*). We interpret this density with an oblong shape at 3.0σ with a 6σ peak height as evidence of a peroxide-derived species. For the X-ray structure of *E. coli* MnSOD that was peroxide-soaked, one of the four chains has a PEO molecule modelled at 0.50 occupancy in a similar orientation (Porta *et al.*, 2010 ▸). As a quick test, a dioxygen species was modelled into the neutron scattering-length density and refined. The model refined well at full occupancy to *B*-factor values well below those of neighbouring atoms, although a more meticulous process of model building and refinement is ongoing. Nonetheless, the evidence in the neutron |*F*_o_| − |*F*_c_| of chain *B* (Fig. 5 ▸ *b*) confirms a model of peroxide being cryotrapped.

## 4. Concluding remarks

Altogether, we present the technical details leading to 2.3 Å resolution neutron data collection from large and cryocooled perdeuterated Trp161Phe MnSOD crystals and the evidence for the cryotrapping of a derivative species of D_2_O_2_ bound at the active site. The methods described in this article have laid the foundation for a completed refined model using data sets from X-ray and neutron diffraction. The *P*6_1_22 crystal system of MnSOD has been challenging to navigate using NPC due to its large unit-cell volume and has been one of the largest to be studied to <2.5 Å resolution, second only to that of the Fenna–Matthews–Olson photosynthetic antenna complex (Lu *et al.*, 2019 ▸). To our knowledge, the unit-cell volume of *P*6_1_22 MnSOD is the largest to be studied by cryogenic neutron analysis to <2.5 Å resolution, where hydrogen positions can be observed (Meilleur *et al.*, 2018 ▸). The circumstances prior to data collection required the development of a process that was capable of nearly full deuterium exchange and cryoprotectant acclimatization in hours with minimal damage to the crystal (Fig. 1 ▸). Ostensibly, the simple process can be adapted and applied to other cases where the content of the mother liquor should be changed. Our preliminary neutron refinement to measure the exchange of backbone amide protons estimated 83% deuteration (Fig. 4 ▸) and compares well with investigations that utilized much longer exchange timeframes or crystallized proteins with deuterated reagents (Kwon *et al.*, 2016 ▸; Li *et al.*, 2017 ▸; Dajnowicz *et al.*, 2017 ▸). We also describe our methods for cryocooling large MnSOD crystals for NPC (Figs. 2 ▸ and 3 ▸), where a crystal can be picked up from its drop and undergo vitreous freezing on the goniometer within seconds. D_2_O_2_ was soaked into a 0.65 mm^3^ perdeuterated Trp161Phe MnSOD crystal prior to cryocooling, which led to data indicating a D_2_O_2_-derivative species bound to the manganese at the most solvent-accessible active site (Fig. 5 ▸). The visualization of a putative oxygen-ligand species bound to a paramagnetic metal centre without radiation-induced structural change is especially noteworthy for studies of oxidoreductases.

## Supplementary Material

Preliminary refinement data.: https://doi.org/10.5281/zenodo.5715407

## Figures and Tables

**Table 1:** Data-collection statistics for perdeuterated Trp161Phe MnSOD crystals

	Neutron	X-ray
Ligand state	D_2_O_2_-soaked	Apo
Crystal volume (mm^3^)	0.65	<0.10
Diffraction source	SNS MaNDi	Rigaku FR-E^+^ SuperBright
Temperature (K)	100	100
Space group	*P*6_1_22	*P*6_1_22
*a*, *b*, *c* (Å)	77.8, 77.8, 236.8	78.4, 78.4, 237.0
α, β, γ (°)	90, 90, 120	90, 90, 120
Wavelength(s) (Å)	2–4	1.5418
Detector(s)	40 SNS Anger cameras	R-AXIS IV++
Crystal-to-detector distance (mm)	450	240
Rotation range between images (°)	10	—
Rotation range per image (°)	0	0.5
Total rotation range (°)	120	126
No. of images collected	12	252
No. of unique reflections	19467	34472
Total No. of reflections	189145	224426
Resolution range (Å)	14.82–2.30 (2.38–2.30)	50.0–1.87 (1.91–1.87)
Completeness (%)	99.01 (98.04)	93.8 (89.3)
Multiplicity	9.7 (7.0)	6.5 (5.2)
〈*I*/σ(*I*)〉	9.7 (5.6)	11.4 (2.0)
*R*_merge_	0.279 (0.286)	—
*R*_meas_	0.294 (0.306)	0.12 (0.76)
*R*_p.i.m._	0.086 (0.102)	0.044 (0.331)
